# Tea Consumption and New-Onset Acute Kidney Injury: The Effects of Milk or Sweeteners Addition and Caffeine/Coffee

**DOI:** 10.3390/nu15092201

**Published:** 2023-05-05

**Authors:** Mengyi Liu, Sisi Yang, Ziliang Ye, Yanjun Zhang, Yuanyuan Zhang, Panpan He, Chun Zhou, Fan Fan Hou, Xianhui Qin

**Affiliations:** Division of Nephrology, Nanfang Hospital, Southern Medical University, National Clinical Research Center for Kidney Disease, State Key Laboratory of Organ Failure Research, Guangdong Provincial Institute of Nephrology, Guangdong Provincial Key Laboratory of Renal Failure Research, Guangzhou 510515, China

**Keywords:** tea, acute kidney injury, caffeine metabolism, tea additives, coffee

## Abstract

**Aims:** To explore the relationship between tea consumption and the risk of incident acute kidney injury (AKI) and examine the effects of coffee consumption, genetic variation in caffeine metabolism, and the use of tea additives (milk and sweeteners) on this association. **Methods:** Using data from the UK Biobank, 498,621 participants who were free of AKI and had information on tea intake were included. Black tea is the main type consumed in this population. Dietary information was collected from standardized and validated Food-Frequency Questionnaire (FFQ). Outcome was incident AKI, determined via primary care data, hospital inpatient data, death register records, or self-reported data at follow-up visits. **Results:** After a median follow-up period of 12.0 years, 21,202 participants occurred AKI. Overall, there was a reversed J-shaped relation between tea consumption and incident AKI, with an inflection point at 3.5 cup/d (*p* for nonlinearity < 0.001). The relation was similar among participants with different genetically predicted caffeine metabolism (*p*-interaction = 0.684), while a more obvious positive association was found between heavy tea consumption and AKI when more coffee was consumed (*p*-interaction < 0.001). Meanwhile, there was a reversed J-shaped relationship for drinking tea with neither milk nor sweeteners, and a L-shaped association for drinking tea with milk (with or without sweeteners) with incident AKI. However, no significant association was found between drinking tea with sweeteners only and incident AKI. **Conclusions:** There was a reversed J-shaped relation between tea consumption and incident AKI, suggesting that light to moderate tea consumption, especially adding milk, can be part of a healthy diet.

## 1. Introduction

Acute kidney injury (AKI), formerly known as acute renal failure (ARF), is a major public health concern occurring in approximately 13.3 million persons each year [1]. AKI is associated with both short- and long-term risks of adverse outcomes, including end-stage kidney disease (ESKD), prolonged hospitalization, reduced quality of life, and mortality [2,3,4]. As such, it is essential to understand and identify more modifiable risk factors of AKI in a community setting.

AKI is a clinical syndrome characterized by a sudden decline in renal excretory function and resultant accumulation of metabolic waste products. The pathophysiology of AKI is multifaceted and usually associated with a focal mismatch between impaired delivery of oxygen and nutrients to the nephron and incremental renal energy and oxygen requirements [5,6]. Additionally, inflammation and oxidative stress are two important mechanisms of AKI. Considering the link between diet and immune function [7], it is speculated that dietary modification, especially antioxidants, may alter the susceptibility to AKI [8]. Consistently, the Atherosclerosis Risk in Communities (ARIC) study found that higher coffee consumption, which has anti-inflammatory properties, was related with a lower hazard of new-onset AKI, suggesting a possibility for cardiorenal protection through diet [9]. Tea is another beverage most commonly consumed in the United Kingdom and worldwide. Tea is extracted from the leaves of the plant Camellia sinensis, and contains a variety of biomolecules, including flavonoids and other antioxidants, all of which exhibit biological activities such as free radical scavenging and antioxidant properties [10]. Growing evidences from animals have shown that tea has a protective effect on kidney damage caused by high-fat diet, ischemia-reperfusion, proline, lead, or gentamicin by inhibiting inflammation and cell apoptosis, decreasing lipid peroxidation in the kidney, scavenging reactive oxygen species (ROS), and alleviating autophagy suppression [11]. Nevertheless, although an association between excessive iced tea consumption and acute kidney failure was reported in one case report [12], to date, few studies have investigated the prospective relation between tea intake and AKI.

Most important, tea also contains caffeine, and is a major contributor to dietary caffeine, though it is usually less caffeinated than coffee [13]. Given the growing concerns regarding high caffeine intake, particularly among individuals with slow caffeine metabolism [14,15,16], exploring the modifying effects of genetic variation in caffeine metabolism and coffee intake on the relationship of tea intake with AKI may yield new insights for clinical and public health. At the same, whether the common additives in tea, such as milk and sweeteners, may affect the relation between tea consumption and AKI also remains uncertain.

As such, the present study aimed to explore the dose-response association of tea consumption with new-onset AKI in the UK Biobank, a cohort study including half a million participants living in the United Kingdom where black tea consumption is very common. Furthermore, we also evaluated the potential modifying effects of coffee consumption, genetic variation in caffeine metabolism and use of common additives such as milk and sweeteners on the association.

## 2. Methods

### 2.1. Data Source and Study Population

As previously described [17,18], the community-based UK Biobank is a large prospective, observational, population-based cohort recruiting half a million volunteers, aged 37–73 years, from 1 of 22 assessment centers across the United Kingdom (England, Wales, and Scotland) during 2006 and 2010. Participants completed a comprehensive questionnaire survey using a touch screen at assessment center to collect information related to sociodemographic, lifestyle, and health. They underwent physical examinations and provided biological samples for laboratory analysis. Moreover, hospital inpatient records, death certificates and primary care records were linked to determine clinical outcomes. The UK Biobank was approved by the North West Research Ethics Committee (06/MRE08/65) and all participants signed an informed consent.

In this study, participants who had complete information on tea and coffee consumption were included. Those with AKI diagnosis prior to date of baseline assessment were further excluded. Therefore, a total of 498,621 participants were enrolled in the present analysis (Supplemental Figure S1).

### 2.2. Dietary Assessment

Dietary information was collected via standardized and validated touchscreen Food-Frequency Questionnaire (FFQ) completed by the study participants at baseline. For tea and coffee, participants were asked how many cups of tea (include black and green tea) or coffee (include decaffeinated coffee) they drank each day in the last year. Participants would be asked to confirm their response when they reported drinking more than 20 cups tea or 10 cups coffee per day. Among 20,348 participants who repeated the baseline touchscreen questionnaire approximately 4 years after recruitment, the weighted κ of tea intake across the 2 assessments was 0.83 [19,20]. A healthy diet score was defined according to a more recent dietary recommendation for cardiovascular health, which considered adequate intake of fruit, vegetables, whole grains, fish, shellfish, dairy products, and vegetable oils; and reduced intake of refined grains, processed meats, unprocessed meats, and sugar sweetened beverages [21].

Information regarding use of tea additives was not available in the baseline touchscreen questionnaire; instead, in a subset of 70,699 participants, corresponding questions were investigated by a 24-h dietary recall questionnaire from 2009 to 2010. Furthermore, four additional 24-h dietary recall questionnaires were emailed between 2011 and 2012. In each 24-h dietary recall, if participants drank tea, they were asked whether they added milk or sweeteners (including sugar and/or artificial sweetener) to their tea. Overall, 210,313 participants completed at least one of the five 24-h dietary recall questionnaires. 18,688 participants with inconsistent tea consumption status between the 24-h dietary recall questionnaire and baseline touchscreen FFQ (e.g, participants reporting tea consumption in FFQ but no tea consumption in the 24-h dietary recall questionnaire) were further excluded, and therefore, 191,625 participants were included in the analysis. We classified the addition of milk and/or sweeteners to tea in any of the dietary recalls as adding milk and/or sweetener to tea, respectively, and the absence of milk and/or sweeteners to tea in all of the dietary recalls as not using tea additives. Accordingly, tea consumers were divided into four groups: consumers who added neither milk or sweeteners, added only milk, added only sweeteners, and added both milk and sweeteners to tea. 

### 2.3. Covariates Measurements

Detailed information regarding covariates was obtained via several touch-screen computer-based questionnaires, and face-to-face interviews with trained researchers. The information collected included demographics (sex and age), ethnicity, socioeconomic factors (educational attainment, household income, employment, Townsend Deprivation Index [TDI]), behavioural factors (smoking status, alcohol consumption, and moderate and vigorous physical activity), and comorbidities (diabetes, hypertension, high cholesterol, cardiovascular disease [CVD] and chronic kidney disease [CKD]). Optimal physical activity was defined as at least 4 days of vigorous/moderate physical activity in a typical week. Estimated glomerular filtration rate (eGFR) was calculated by Chronic Kidney Disease Epidemiology Collaboration equation [22]. 

### 2.4. Genetic Caffeine Metabolism Score

Detailed information on genotyping and quality control in the UK Biobank has been previously described [23]. Based on four common single-nucleotide polymorphisms (rs2472297, rs56113850, rs6968554, and rs17685) previously associated with blood caffeine metabolite levels and located in or near genes involved in caffeine metabolism, the weighted genetic caffeine metabolism score (wCMSG4) was calculated via summing the number of alleles multiplied by their β-coefficients [19]. Four categories were created for wCMSG4 based on the quartiles of wCMSG4, and a higher score indicated faster caffeine metabolism.

### 2.5. Ascertainment of AKI

Incident AKI was defined based on the International Classification of Diseases edition 10 (ICD-10) code N17, including N17.0 for ARF with tubular necrosis, N17.1 for ARF with acute cortical necrosis, N17.2 for ARF with medullary necrosis, N17.8 for other ARF and N17.9 for unspecified ARF, ascertained by primary care data, hospital inpatient data, death register records, or self-reported medical conditions at follow-up visits. A previous study conducted in the UK examined the validity of ICD-10 N17 codes against the KDIGO criteria for AKI occurrence, and estimated a positive predictive value of 95% for the ICD-10 codes of N17 [24]. Renal replacement therapy was ascertained by Office of Population Censuses and Surveys Classification of Interventions and Procedures (OPCS) code (Supplemental Table S1). More details about the ascertainment of the outcome can be found online (https://biobank.ndph.ox.ac.uk/showcase/label.cgi?id=1712 (accessed on 20 March 2023)). The follow-up time was calculated from the date of initial assessment until the date of death, first date of AKI diagnosis, date of loss of follow-up, or end of follow-up, whichever came first. 

## 3. Statistical Analysis

Comparison of baseline characteristics according to tea consumption was performed by chi-square test and analysis of variance for categorical and continuous variables, respectively.

Restricted cubic spline (RCS) Cox regression was performed to test for linearity and explore the shape of the dose-response relation of tea consumption and incident AKI. Cox proportional hazards models were used to estimate hazard ratio (HR) and 95% confidence interval (CI) of AKI for tea consumption with no tea consumption as the referent group. In multivariable models, potential confounders that were known to be traditional or suspected risk factors for renal disease were adjusted for, including sex, age, ethnicity, educational attainment, household income, employment, TDI, body mass index (BMI), smoking status, alcohol consumption, physical activity, healthy diet scores, coffee consumption, water consumption, and comorbidities (diabetes, hypertension, high cholesterol, CVD and CKD). Furthermore, among 191,625 participants who completed at least one 24-h dietary recall questionnaire and had consistent tea consumption status between 24-h dietary recall questionnaire and baseline touchscreen FFQ, we investigated the joint effect of tea consumption and the addition of milk and/or sweeteners to tea with new-onset AKI by examining the association between tea consumption and new-onset AKI in tea consumers with different additives (milk and/or sweeteners) with no tea consumption as the referent group. 

To evaluate the potential modification effects of caffeine, stratified analyses were performed according to genetically predicted caffeine metabolism (quartiles of wCMSG4) and coffee consumption (0, ≤2, 3–5, ≥6 cup per day). As additional exploratory analyses, other possible modifications were also assessed for variables including age, sex, BMI, smoking status, alcohol consumption, diabetes, hypertension, high cholesterol (yes or no), CVD, CKD, healthy diet scores, and optimal physical activity. Potential modifying effects were evaluated by modelling the cross-product term between the stratifying variable and tea consumption.

We also performed a series of sensitivity analyses. First, we further adjusted for intensive care unit (ICU) admission, sepsis, viral infections, and bacterial infections before the end of follow-up, which were obtained via hospital inpatient records. Second, we further adjusted for drug uses (cholesterol lowering medication, anti-hypertensive drug, antidiabetic, aspirin, ibuprofen, paracetamol, and diclofenac) at baseline. Third, since subjects with migraines can have endothelial dysfunction and the role of caffeine maybe protective, we further excluded participants with a previous history of migraines and defined the new migraines diagnosis during follow-up as censored data. Fourth, we further adjusted for insomnia and irritability, which may be influenced by caffeine. Insomnia was collected by asking “Do you have trouble falling asleep at night or do you wake up in the middle of the night?”, and irritability was obtained by asking “Are you an irritable person?”. Fifth, we further excluded participants with a previous history of drug use disorders and defined the new drug use disorders diagnosis during follow-up as censored data. Sixth, a subset of participants was asked about their life-time cannabis use by ‘Have you taken cannabis (marijuana, grass, hash, ganja, blow, draw, skunk, weed, spliff, dope), even if it was a long time ago?’. We further adjusted for lifetime cannabis use. Seventh, we restricted the analyses on a subsample of participants who reported no any major diet changes in the last 5 years. Eighth, we explored the dose-response relationship of caffeine consumption and new-onset AKI. Caffeine intake was calculated by multiplying the number of cups of coffee or tea by the content of caffeine per cup. Decaffeinated coffee was considered to contain 3 mg of caffeine per cup, instant coffee 60 mg, ground coffee 85 mg, and tea 30 mg [25]. Finally, to further validate the results for adding milk to tea, we assessed the joint association of tea consumption and milk consumption with the risk of AKI.

A two-tailed *p* < 0.05 was considered to be statistically significant in all analyses. Analyses were performed using R 4.1.1 software (http://www.R-project.org/ (accessed on 20 March 2023)).

## 4. Results

### 4.1. Study Participants and Baseline Characteristics

Among 498,621 participants in the present study, 271,543 (54.5%) were females, with a median age of 58.0 (IQR: 50.0–63.0) years. 85.3% of participants reported consuming tea, and most (40.5%) consumed 3 to 5 cups of tea/day. The relations of tea consumption with some potential risk factors were complex (Table 1). For example, moderate tea drinkers (3 to 5 cups/day) were less likely to be current smokers, as well as had lower TDI, BMI and eGFR, than non-tea consumers or heavy tea consumers (that is, ≥9 cups/day). Heavy tea consumers tended to be males, had higher urine albumin-to-creatinine ratio (UACR) and higher prevalence of high cholesterol, CVD and CKD, while non-tea drinkers tended to drink more coffee. 

### 4.2. Association between Tea Consumption and New-Onset AKI

After a median follow-up of 12.0 years, 21,202 (4.3%) participants developed new-onset AKI. 96.2% of AKI cases were ascertained by hospital admissions recodes. Most of the AKI cases were unspecified acute renal failure and non-dialysis-requiring AKI, and only 20 participants developed new-onset AKI requiring renal replacement therapy during hospitalization.

**Figure 1:**
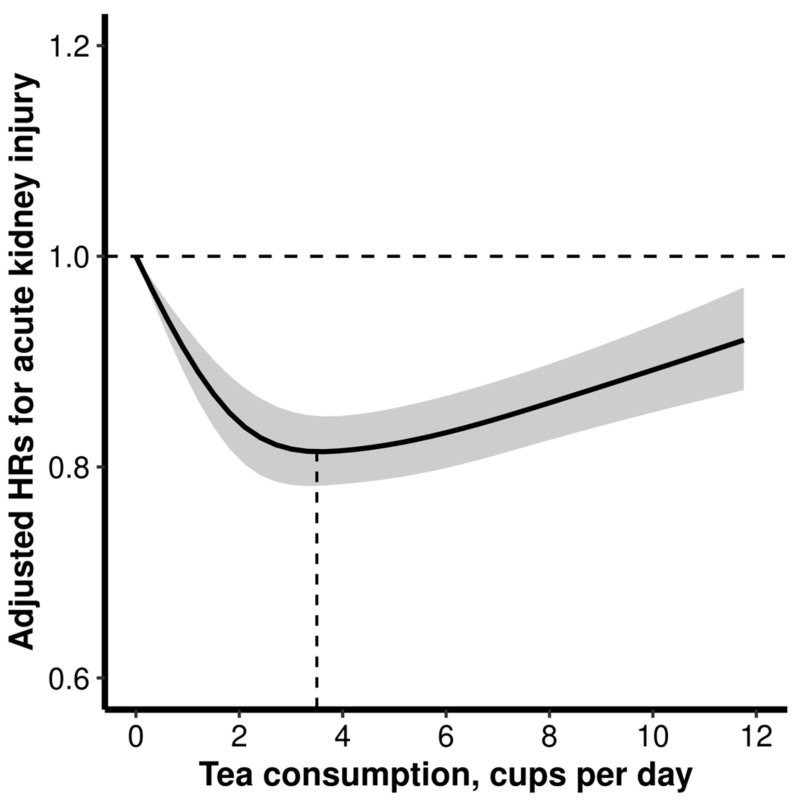
Dose-response relation of tea intake with new-onset acute kidney injury (AKI) *. * Results were adjusted for age, sex, ethnicity, educational attainment, household income, employment, Townsend Deprivation Index, body mass index, smoking status, alcohol consumption, physical activity, healthy diet score, coffee consumption, and comorbidities (hypertension, diabetes, high cholesterol, cardiovascular disease and chronic kidney disease).

Overall, the relation between tea consumption and the hazard of incident AKI followed a reverse J-shape, with an inflection point at 4 cup/d (*p* for nonlinearity < 0.001; Figure 1). Relative to non-tea drinkers, adjusted HRs (95% CIs) of incident AKI for participants who reported drinking 2 or fewer, 3 to 5, 6 to 8, and 9 or more cups of tea per day were 0.87 (0.83–0.91), 0.81 (0.78–0.85), 0.83 (0.79–0.87), and 0.95 (0.89–1.02), respectively (Table 2). Additionally, there was a J-shaped relation of overall coffee consumption with the hazard of incident AKI (Supplemental Figure S2).

### 4.3. Joint Effect of Tea Consumption and Addition of Milk or Sweeteners to Tea on New-Onset AKI 

Among 191,625 participants who completed at least one 24-h dietary recall questionnaire, among tea drinkers, 91.7% of tea drinkers (*n* = 154,418) drank black tea and 10.8% (*n* = 18,211) drank green tea. The association of tea intake with AKI was similar for black tea drinkers and green tea drinkers (Supplemental Table S2).

**Figure 2:**
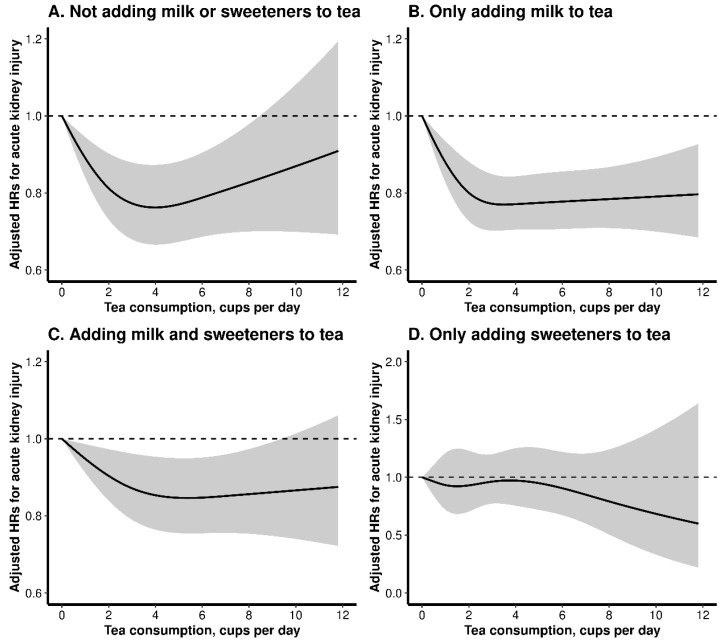
Dose-response relation of tea intake with new-onset acute kidney injury (AKI) stratified by the addition of milk or sweeteners to tea.The analysis was performed in participants who completed at least one 24-h dietary recall questionnaire. Results were adjusted for age, sex, ethnicity, educational attainment, household income, employment, Townsend Deprivation Index, body mass index, smoking status, alcohol consumption, physical activity, healthy diet score, coffee consumption, and comorbidities (hypertension, diabetes, high cholesterol, cardiovascular disease and chronic kidney disease).

Moreover, 14.0% of tea drinkers added neither milk nor sweeteners to tea, while 64.6%, 2.0%, and 19.5% added milk only, sweeteners only, and milk and sweeteners both to tea, respectively. Overall, there was a reversed J-shaped trend between tea consumption and incident AKI among those who added neither milk nor sweeteners to tea (Figure 2A and Supplemental Table S3), while a L-shaped association was found among those who reported added milk to tea, whether adding sweeteners or not (Figure 2B,C and Supplemental Table S3). However, no significant relation was found between tea consumption and new-onset AKI among those who added sweeteners only to tea (Figure 2D and Supplemental Table S3). In the joint analysis of tea and milk intake with the hazard of AKI, a similar L-shaped relation of tea consumption with incident AKI was found among those who consumed both tea and milk (Supplemental Table S4). 

### 4.4. Stratified Analyses by Potential Effect Modifiers

**Figure 3:**
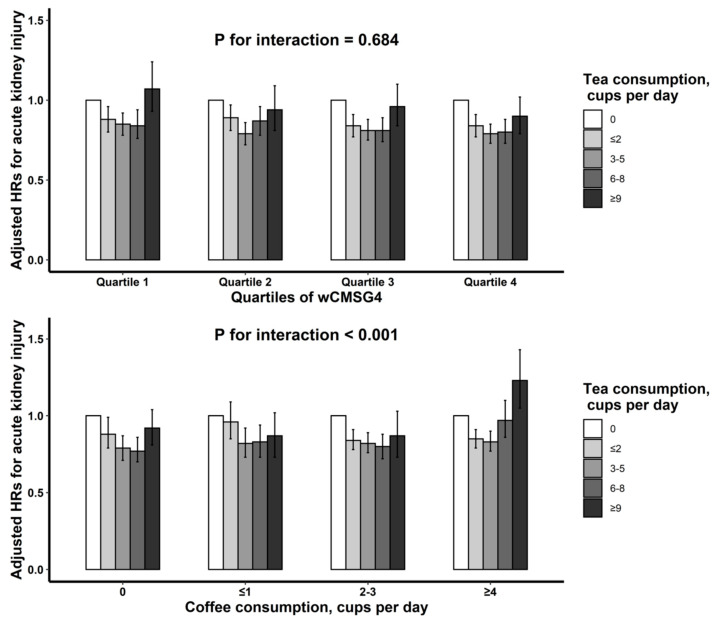
Association of tea consumption with new-onset acute kidney injury (AKI) stratified by genetically predicted caffeine metabolism and coffee consumption. Results were adjusted for age, sex, ethnicity, educational attainment, household income, employment, Townsend Deprivation Index, body mass index, smoking status, alcohol consumption, physical activity, healthy diet score, coffee consumption, and comorbidities (hypertension, diabetes, high cholesterol, cardiovascular disease and chronic kidney disease).

In stratified analyses of genetic scores for caffeine metabolism, similar relations were observed across quartiles of wCMSG4 (*p*-interaction = 0.684; Figure 3). However, a more obvious positive association between heavy tea consumption and AKI was observed among those with higher intake level of coffee (*p* for interaction < 0.001; Figure 3). 

None of the other variables showed significant effect modification on the tea consumption-AKI relation (all *p* for interaction > 0.05; Supplemental Table S5).

### 4.5. Sensitivity Analyses

The results did not change substantially by further adjusting for ICU admission, sepsis, viral infections, and bacterial infections before the end of follow-up (Sensitivity analysis 1); further adjusting for drug uses at baseline (Sensitivity analysis 2); excluding 15,314 participants with a previous history of migraines (Sensitivity analysis 3); further adjusting for insomnia, and irritability (Sensitivity analysis 4); excluding 297 participants with a previous history of drug use disorders (Sensitivity analysis 5); or further adjusting for lifetime cannabis use (Sensitivity analysis 6); or restricting to a subsample without any major diet changes in the last 5 years (Sensitivity analysis 7) (Supplemental Table S6). Furthermore, there was a reverse J-shaped relation of caffeine consumption with incident AKI (Supplemental Figure S2).

## 5. Discussion

In this large-scale prospective study, we firstly observed a reversed J-shaped relation of tea intake with the hazard of new-onset AKI, with the inflection points at 3.5 cup/d. The association was similar among participants with different genetically predicted caffeine metabolism, while a more obvious positive association with heavy tea consumption were found when drinking more coffee. Meanwhile, compared with nontea drinkers, moderate tea drinkers who did not use tea additives or added milk to tea had a significantly lower hazard of new-onset AKI, but no significant risk reduction was found among those who added sweeteners only to tea.

A previous study conducted in renal transplant recipients reported that short-term intake of black tea improved arterial vasodilation and endothelial function [26]. Another study demonstrated that green tea extract could reduce hemodialysis-induced ROS and alleviated the subsequent adverse events [27]. Furthermore, higher tea intake was found to be related with a lower likelihood of albuminuria and increased levels of estimated eGFR [28]. Nevertheless, the reno-protection effect of tea was not previously examined in the setting of AKI. Our study used a large cohort of middle-aged adults with a wide range of regular tea intake, providing an opportunity to explore the dose-response relationship between tea consumption and AKI.

Our research provides some new insights into this field. First, we observed that there was a reversed J-shaped association of tea intake with incident AKI, with an inflection point at 3.5 cup/d. Consistently, previous studies reported that consumption of tea was associated with favorable outcomes in terms of the hazard of acute or chronic ischemic related diseases [11]. The inverse relation of light to moderate tea intake with new-onset AKI is biologically reasonable. AKI, a syndrome characterized by an abrupt loss of kidney function, is usually associated with a focal mismatch between impaired delivery of oxygen and nutrients to the nephron and incremental renal energy and oxygen requirements, and most of the AKI events are attributed to ischemic injuries [5,6]. Tea and its bioactive components could modulate the function of the renin-angiotensin-aldosterone system, enhance the synthesis of endothelial nitric oxide, increase antioxidant activity and inhibit inflammation, and improve the expression of renal sodium-potassium pump, and therefore, reduce renal oxygen consumption, improve hemodynamic recovery during reperfusion, and decrease the susceptibility to AKI during acute injury in the general population [29,30,31].

Second, genetic capacities for caffeine metabolism did not significantly modify the relation of tea consumption with new-onset AKI, implying that caffeine in tea does not underlie the inverse relationship of moderate tea intake with AKI hazard. However, we observed a weaker trend of heavy tea consumption and AKI risk in those with slower genetically predicted caffeine metabolism. Furthermore, we also found that coffee consumption significantly modified the relation between heavy tea intake and AKI. In participants with higher coffee consumption, the risk of AKI was not decreased, or even increased among heavy tea drinkers. Indeed, high caffeine consumption may increase blood pressure and aggravate the injury of kidney in animal models [32]. It is possible that higher caffeine consumption, by blocking the A1 and A2A adenosine receptors and simultaneously increasing the levels of angiotensin II in the kidney and the activity of the renal renin-angiotensin system, may result in a larger portion of the systemic blood pressure being transmitted to glomeruli, leading to kidney damage [32]. Consistently, we did find a J-shaped relation between caffeine intake and incident AKI and high caffeine intake was related with higher AKI hazard. As such, we hypothesized that the benefits of tea may be offset by the detrimental effects of higher caffeine. In addition, the increased trend of AKI hazard related with higher tea intake among participants with tea intake ≥ 3.5 cup/d may also be partly explained by its relatively high caffeine content [33]. However, further researches are needed to confirm our results and further examine the potential mechanisms.

Third, moderate tea intake was related with a lower hazard of new-onset AKI among participants who added neither milk nor sweeteners to tea, or added milk to tea (with or without adding sweeteners). However, no significant association was found between and new-onset AKI among those who added sweeteners only to tea. Western nations have historically built habits of adding milk and sweeteners into tea. It was speculated that the interaction between tea polyphenols and glucose complexes may lower the antioxidative capacity of tea [34]. In the current study, we did observe that adding sweeteners to tea counteracted the renal benefits of light to moderate tea consumption, and should therefore be used with caution. In contrast, we found that the risk of new-onset AKI was lowered when milk was added to tea. In particular, the addition of milk into tea could offset the increased AKI risk associated with high tea consumption. It’s biologically plausible. Recent studies have found that protein-polyphenol interactions may enhance the anti-inflammatory activity of phenolic compounds [35,36]. Additionally, milk proteins may possess renal-protective functions, such as suppressing the angiotensin converting enzyme and lowering blood pressure, through peptides (casokinins and lactokinins) that have vasoactive properties [37,38,39].

There are some limitations. First, considering the observational nature of the study, residual confounding caused by unmeasured or unknown factors is possible. Second, tea intake was assessed at baseline only, and dietary changes over time might be not captured. However, the concordance of tea consumption was high (κ_weighted_ = 0.83) among the subsample of participants who had repeated information of tea consumption at approximately 4 years after recruitment, and the correlation in tea consumption was also good (correlation coefficient r = 0.81) between the baseline touchscreen dietary questionnaire and the mean tea consumption of 24-h dietary recall questionnaires in a subsample of participants who completed at least two 24-h dietary recall questionnaires [19,20,40]. Third, repeated serum and urinary data/parameters (e.g., blood urea nitrogen) were not available in the UK Biobank, and therefore, consistent with previous studies [9,41,42,43,44], the current study used ICD codes to define study outcomes. As such, the AKI onset may be undercounted because less severe AKI cases may not be identified and diagnosed by the physician and, therefore, not coded [45]. However, previous study has indicated that the ICD codes could accurately identify AKI with a very high specificity [24,46]. Fourth, in our study, the only measure of AKI severity was renal replacement therapy, and almost all AKI cases were non-dialysis-requiring AKI. Because serum creatinine and urea nitrogen measurements were not available, we were unable to further grade the severity of non-dialysis-requiring AKI. Fifth, dietary information was obtained from standardized and validated touchscreen FFQ reporting frequency of intake of common foods and beverages over the past year. However, we observed a similar association of tea consumption and new-onset AKI in participants who reported no any major diet changes in the last 5 years. Sixth, we did not have detailed information about urination frequency which may be affected by caffeine. Overall, it is essential to further confirm our results in future research.

## 6. Conclusions

In conclusion, this large-scale prospective study showed a reversed J-shaped relation of tea consumption with the AKI hazard, with an inflection point at 3.5 cup/d. The elevated AKI hazard related with heavy tea consumption was more pronounced in individuals with more coffee consumption. Moreover, moderate tea drinkers who added neither milk nor sweeteners to tea or added milk to tea (with or without adding sweeteners) had a significantly lower AKI hazard, but no significant risk reduction was found among those who added sweeteners only to tea. These novel findings have significant clinical and public health implications. Tea is the second most consumed beverage next to water. As such, even a small benefit to renal health could have a considerable impact on public health. Our data suggest that light to moderate consumption of black tea, especially adding milk, can be part of a healthy diet and may be a relatively simple and low-cost solution to prevent AKI hospitalizations or procedures.

## Figures and Tables

**Table 1:** Baseline population characteristics according to tea consumption categories *.

	Tea Consumption, Cups per Day					*p* Value
	0	≤2	3–5	6–8	≥9	
*n*	73,287	129,272	201,748	74,811	19,503	
Age, years	56.0 (48.0, 62.0)	57.0 (49.0, 63.0)	58.0 (51.0, 64.0)	58.0 (51.0, 63.0)	57.0 (50.0, 63.0)	<0.001
Male, No. (%)	31,880 (43.5)	61,121 (47.3)	90,155 (44.7)	33,677 (45.0)	10,245 (52.5)	<0.001
White, No. (%)	69,847 (95.7)	117,837 (91.5)	191,124 (95.0)	72,834 (97.7)	18,842 (97.0)	<0.001
TDI	−1.9 (−3.5, 0.9)	−2.1 (−3.6, 0.7)	−2.3 (−3.7, 0.2)	−2.2 (−3.6, 0.4)	−1.5 (−3.3, 1.6)	<0.001
Education, No. (%)						<0.001
No secondary education	12,085 (16.5)	16,970 (13.1)	35,015 (17.4)	15,275 (20.4)	4799 (24.6)	
Secondary education	37,907 (51.7)	61,523 (47.6)	98,751 (48.9)	37,378 (50.0)	9247 (47.4)	
University degree	22,059 (30.1)	48,481 (37.5)	64,305 (31.9)	20,785 (27.8)	5075 (26.0)	
Income, No. (%)						<0.001
Less than £31,000	30,284 (41.3)	48,223 (37.3)	83,094 (41.2)	32,989 (44.1)	9406 (48.2)	
At least £31,000	43,003 (58.7)	81,049 (62.7)	118,654 (58.8)	41,822 (55.9)	10,097 (51.8)	
Employed, *n* (%)	66,482 (91.5)	119,284 (93.3)	187,003 (93.5)	68,641 (92.5)	16,894 (87.7)	<0.001
BMI, kg/m^2^	27.4 (24.5, 30.9)	26.6 (24.0, 29.8)	26.5 (24.0, 29.6)	26.8 (24.2, 29.9)	27.0 (24.3, 30.2)	<0.001
Smoking status, No. (%)						<0.001
Never	38,105 (52.2)	71,481 (55.5)	113,761 (56.6)	40,103 (53.8)	8705 (44.8)	
Former	24,924 (34.1)	44,764 (34.7)	70,380 (35.0)	25,713 (34.5)	6420 (33.1)	
Current	10,029 (13.7)	12,573 (9.8)	16,848 (8.4)	8688 (11.7)	4296 (22.1)	
Alcohol consumption, times/week						<0.001
<1	25,784 (35.2)	35,462 (27.5)	58,151 (28.8)	25,568 (34.2)	8023 (41.2)	
1–2	17,602 (24.0)	31,367 (24.3)	54,409 (27.0)	20,481 (27.4)	4829 (24.8)	
3–4	14,616 (20.0)	31,018 (24.0)	49,164 (24.4)	16,615 (22.2)	3662 (18.8)	
>4	15,223 (20.8)	31,312 (24.2)	39,894 (19.8)	12,084 (16.2)	2963 (15.2)	
Physical activity, days/week						<0.001
Moderate	3.0 (2.0, 5.0)	3.0 (2.0, 5.0)	3.0 (2.0, 5.0)	4.0 (2.0, 6.0)	4.0 (2.0, 6.0)	
Vigorous	1.0 (0.0, 3.0)	1.0 (0.0, 3.0)	1.0 (0.0, 3.0)	1.0 (0.0, 3.0)	1.0 (0.0, 3.0)	
Healthy diet score	3.0 (2.0, 4.0)	3.0 (2.0, 4.0)	3.0 (2.0, 4.0)	3.0 (2.0, 4.0)	3.0 (2.0, 4.0)	<0.001
Coffee consumption, cups per day	3.0 (1.0, 5.0)	2.0 (1.0, 4.0)	1.0 (0.5, 2.0)	1.0 (0.0, 2.0)	0.5 (0.0, 2.0)	<0.001
Water consumption, glasses per day	3.0 (1.0, 5.0)	3.0 (1.0, 4.0)	2.0 (1.0, 4.0)	2.0 (1.0, 3.0)	2.0 (0.5, 3.0)	<0.001
Disease history, No. (%)						
Diabetes	5102 (7.5)	7767 (6.5)	10,871 (5.8)	3994 (5.7)	1269 (7.0)	<0.001
Hypertension	40,274 (55.5)	70,644 (55.1)	112,528 (56.3)	41,830 (56.4)	10,847 (56.2)	<0.001
High cholesterol	13,805 (19.1)	23,656 (18.6)	37,457 (18.8)	13,987 (19)	3888 (20.3)	<0.001
CVD	5179 (7.1)	8062 (6.3)	13,722 (6.8)	5603 (7.5)	1813 (9.3)	<0.001
CKD	5508 (8.3)	9228 (7.8)	15,376 (8.4)	5975 (8.7)	1736 (9.8)	<0.001
Creatinine, mg/dL	0.78 (0.68, 0.90)	0.79 (0.69, 0.91)	0.80 (0.70, 0.92)	0.80 (0.70, 0.92)	0.82 (0.71, 0.94)	<0.001
eGFR, mL/min/1.73 m^2^	94.5 (85.1, 102)	93.6 (84.2, 101)	92.1 (82.1, 99.2)	91.8 (81.5, 98.8)	92.5 (82.1, 99.9)	<0.001
eGFR < 60 mL/min/1.73 m^2^, No. (%)	1320 (1.9)	2373 (2.0)	4420 (2.4)	1764 (2.5)	488 (2.7)	<0.001
UACR, mg/g	7.2 (4.6,12.2)	7.2 (4.6,12.1)	7.8 (4.9,12.8)	8.4 (5.2,13.6)	8.8 (5.4,14.7)	<0.001
UACR ≥ 30 mg/g, No. (%)	3882 (5.5)	6463 (5.2)	10,072 (5.2)	3867 (5.4)	1200 (6.4)	<0.001

* Values are expressed in median (interquartile range [IQR]) or proportions. Abbreviations: BMI, body mass index; CKD, chronic kidney disease; CVD, cardiovascular disease; eGFR, estimated glomerular filtration rate; SBP, systolic blood pressure; TDI, Townsend Deprivation Index; UACR, urine albumin-to-creatinine ratio.

**Table 2:** Association of tea intake with the hazard of new-onset acute kidney injury (AKI).

Tea Intake, Cups per Day	Total	No of Events	Incidence Rates *	Crude Model		Adjusted Model 1 ^†^		Adjusted Model 2 ^†^	
				HR (95% CI)	*p* Value	HR (95% CI)	*p* Value	HR (95% CI)	*p* Value
Category									
0	73,287	3638	4.3	ref		ref		ref	
≤2	129,272	5253	3.5	0.81 (0.78, 0.84)	<0.001	0.73 (0.70, 0.76)	<0.001	0.87 (0.83, 0.91)	<0.001
3–5	201,748	8040	3.4	0.80 (0.76, 0.83)	<0.001	0.68 (0.65, 0.71)	<0.001	0.81 (0.78, 0.85)	<0.001
6–8	74,811	3192	3.7	0.86 (0.82, 0.90)	<0.001	0.75 (0.71, 0.78)	<0.001	0.83 (0.79, 0.87)	<0.001
≥9	19,503	1079	4.8	1.14 (1.06, 1.22)	<0.001	1.00 (0.94, 1.07)	0.947	0.95 (0.89, 1.02)	0.150

* Incidence rates per 1000 person years. ^†^ Adjusted for age, sex, and ethnicity in Adjusted Model 1; adjusted for the covariates in Model 1 and further adjusted for educational attainment, household income, employment, Townsend Deprivation Index, body mass index, smoking status, alcohol consumption, physical activity, healthy diet score, coffee consumption, water consumption, and comorbidities (hypertension, diabetes, high cholesterol, cardiovascular disease and chronic kidney disease) in Adjusted Model 2.

## Data Availability

The UK Biobank data are available on application to the UK Biobank, and the analytic methods, and study materials that support the findings of this study will be available from the corresponding authors on request.

## Supplementary Materials

The following supporting information can be downloaded at: https://www.mdpi.com/article/10.3390/nu15092201/s1, Figure S1: Flow chart of the participants in the current analysis; Figure S2: Dose-response association of coffee and caffeine consumption and new-onset acute kidney injury (AKI); Table S1: Disease definitions used in the UK Biobank study; Table S2: The relationship of tea consumption with the risk of new-onset acute kidney injury (AKI) stratified by tea types; Table S3: The relationship of tea consumption with risk of new-onset acute kidney injury (AKI) stratified by added milk or sweeteners in tea; Table S4: The joint relationship of tea consumption and milk consumption with risk of new-onset acute kidney injury (AKI); Table S5: HR (95% CI) for new-onset acute kidney injury (AKI) associated with tea consumption in various subgroups; Table S6: Sensitivity analysis for the association between tea consumption and new-onset acute kidney injury.
