# Supplementary material for: Tea Consumption and New-Onset Acute Kidney Injury: The Effects of Milk or Sweeteners Addition and Caffeine/Coffee
Source: Nutrients. 2023 May 5;15(9):2201. doi: 10.3390/nu15092201 (PMC10180691; doi:10.3390/nu15092201)
Supplement: Supplementary file 1 [file nutrients-15-02201-s001.zip › nutrients-2339648-supplementary.pdf]

List of supplemental materials

Supplemental Figure S1. Flow chart of the participants in the current analysis.

Supplemental Figure S2. Dose-response association of coffee and caffeine consumption and new-onset acute kidney injury (AKI).

Supplemental Table S1. Disease definitions used in the UK Biobank study.

Supplemental Table S2. The relationship of tea consumption with the risk of new-onset acute kidney injury (AKI) stratified by tea types.

Supplemental Table S3. The relationship of tea consumption with risk of new-onset acute kidney injury (AKI) stratified by added milk or sweeteners in tea.

Supplemental Table S4. The joint relationship of tea consumption and milk consumption with risk of new-onset acute kidney injury (AKI).

Supplemental Table S5. HR (95%CI) for new-onset acute kidney injury (AKI) associated with tea consumption in various subgroups.

Supplemental Table S6. Sensitivity analysis for the association between tea consumption and new-onset acute kidney injury.

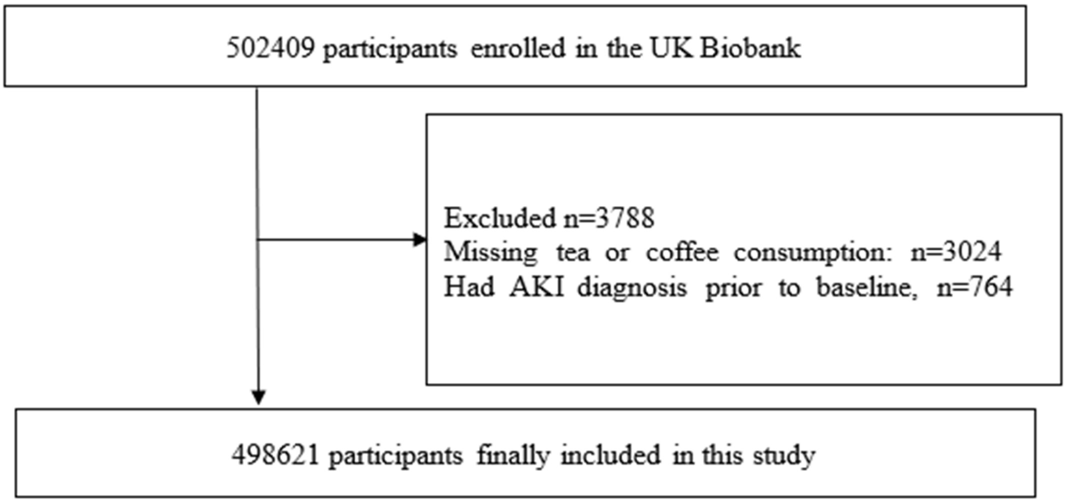

Supplemental Figure S1. Flow chart of the participants in the current analysis.

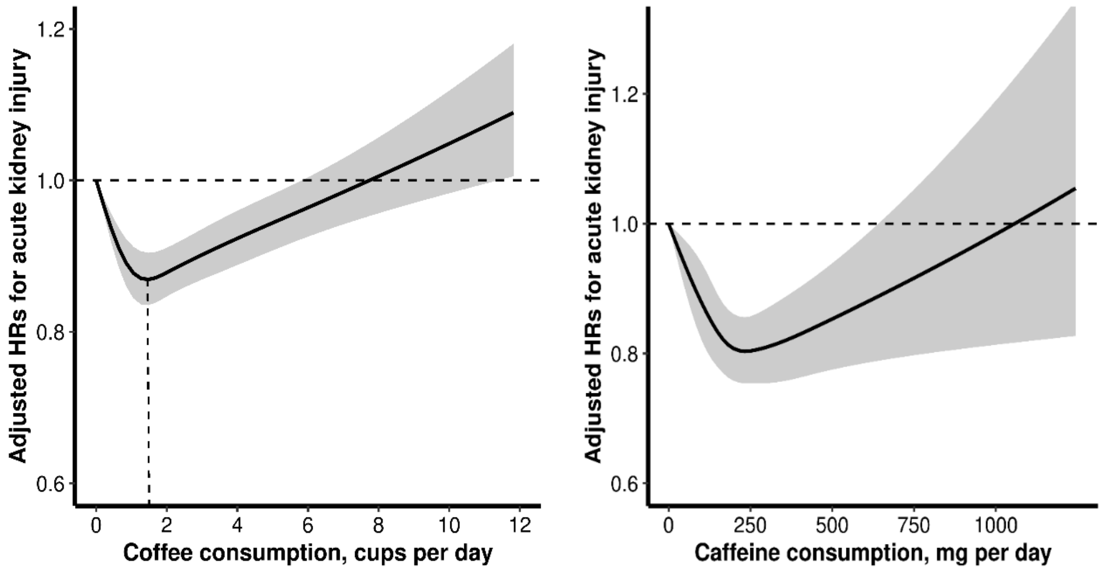

Supplemental Figure S2. Dose-response association of coffee and caffeine consumption with new-onset acute kidney injury (AKI)*.

*Results were adjusted for age, sex, ethnicity, educational attainment, household income, employment, Townsend Deprivation Index, body mass index, smoking status, alcohol consumption, physical activity, healthy diet score, coffee consumption (only for caffeine consumption), tea consumption, and comorbidities (hypertension, diabetes, high cholesterol, cardiovascular disease and chronic kidney disease).

Supplemental Table S1. Disease definitions used in the UK Biobank study.

Disease	ICD-10	OPCS-4
Acute kidney injury	N17.0, N17.1, N17.2, N17.8, N17.9	
Renal replacement therapy		M01.2, M01.3, M01.4, M01.5, M01.8, M01.9, M08.4, M17.2, M17.4, M17.8, M17.9, X40.1-X40.9, X41.1, X41.2, X41.8, X41.9, X42.1, X42.8, X42.9, X43.1
"Disease manifestation" code of acute kidney injury	I12.0, I13.9, K76.7, N05.5, N05.6, N05.9, N13.9, N17.0, N17.1, N17.2, N17.8, N17.9, N19, N99.0, P96.0, R39.2, T79.5	

Abbreviations: ICD, International Classification of Diseases; OPCS, the Office of Population Censuses and Surveys Classification of Interventions and Procedures.

Supplemental Table S2. The relationship of tea consumption with the risk of new-onset acute kidney injury (AKI) stratified by tea types*.

Tea consumption, cups per day	Total	No of events	Adjusted Model 1†		Adjusted Model 2†	
			HR (95%CI)	*P value*	HR (95%CI)	*P value*
**0**	23267	924	ref		ref	
**Black tea**						
≤2	42424	1230	0.64(0.59, 0.70)	<0.001	0.82(0.75, 0.90)	<0.001
3-5	77574	2253	0.64(0.59, 0.69)	<0.001	0.82(0.75, 0.89)	<0.001
6-8	27833	795	0.65(0.59, 0.72)	<0.001	0.78(0.70, 0.86)	<0.001
≥9	6587	226	0.80(0.69, 0.93)	0.003	0.83(0.71, 0.96)	0.014
**Green tea**						
≤2	5538	125	0.53(0.44, 0.64)	<0.001	0.71(0.59, 0.87)	0.001
3-5	9079	222	0.56(0.48, 0.65)	<0.001	0.78(0.66, 0.92)	0.003
6-8	2896	62	0.52(0.40, 0.68)	<0.001	0.71(0.55, 0.93)	0.013
≥9	698	11	0.40(0.22, 0.72)	0.002	0.47(0.26, 0.85)	0.013

* The analysis was performed in 191,625 participants who completed at least one 24-hour dietary recall questionnaire, and had consistent tea consumption status between 24-hour dietary recall questionnaire and baseline touchscreen questionnaire.

† Adjusted Model 1: Adjusted for age, sex, and ethnicity; Adjusted Model 2: adjusted for the covariates in Model 1 and further adjusted for educational attainment, household income, employment, Townsend Deprivation Index, body mass index, smoking status, alcohol consumption, physical activity, healthy diet score, coffee consumption, water consumption, and comorbidities (hypertension, diabetes, high cholesterol, cardiovascular disease and chronic kidney disease).

Supplemental Table S3. The relationship of tea consumption with risk of new-onset acute kidney injury (AKI) stratified by added milk or sweeteners in tea*.

Tea consumption, cups per day	Total	No of events	Adjusted Model 1†		Adjusted Model 2†	
			HR (95%CI)	*P value*	HR (95%CI)	*P value*
**0**	23267	924	ref		ref	
**Not adding milk or sweeteners to tea**						
≤2	9554	245	0.59(0.51, 0.67)	<0.001	0.77(0.67, 0.89)	<0.001
3-5	9964	276	0.60(0.52, 0.69)	<0.001	0.80(0.70, 0.92)	0.002
6-8	3179	87	0.64(0.51, 0.80)	<0.001	0.82(0.66, 1.03)	0.088
≥9	862	31	0.89(0.62, 1.27)	0.509	1.00(0.70, 1.44)	0.987
**Only adding milk to tea**						
≤2	28335	715	0.58(0.52, 0.64)	<0.001	0.77(0.70, 0.86)	<0.001
3-5	55564	1420	0.58(0.53, 0.63)	<0.001	0.78(0.71, 0.85)	<0.001
6-8	20283	509	0.59(0.53, 0.66)	<0.001	0.73(0.65, 0.82)	<0.001
≥9	4554	137	0.72(0.60, 0.86)	<0.001	0.81(0.67, 0.97)	0.021
**Only adding sweeteners to tea**						
≤2	1392	56	0.85(0.65, 1.12)	0.251	0.95(0.73, 1.25)	0.721
3-5	1411	63	0.88(0.68, 1.14)	0.316	0.95(0.73, 1.23)	0.686
6-8	411	20	1.10(0.71, 1.72)	0.668	1.06(0.68, 1.66)	0.786
≥9	102	2	0.41(0.10, 1.65)	0.212	0.43(0.11, 1.70)	0.227
**Adding both milk and sweeteners to tea**						
≤2	9669	377	0.85(0.75, 0.96)	0.008	0.96(0.85, 1.08)	0.483
3-5	15986	624	0.81(0.73, 0.89)	<0.001	0.90(0.81, 1.00)	0.060
6-8	5576	220	0.83(0.72, 0.96)	0.014	0.86(0.73, 1.00)	0.048
≥9	1516	66	0.99(0.77, 1.27)	0.921	0.82(0.64, 1.06)	0.138

* The analysis was performed in 191,625 participants who completed at least one 24-hour dietary recall questionnaire, and had consistent tea consumption status between 24-hour dietary recall questionnaire and baseline touchscreen questionnaire.

†Adjusted Model 1: Adjusted for age, sex, and ethnicity; Adjusted Model 2: adjusted for the covariates in Model 1 and further adjusted for educational attainment, household income, employment, Townsend Deprivation Index, body mass index, smoking status, alcohol consumption, physical activity, healthy diet score, coffee consumption, water consumption, and comorbidities (hypertension, diabetes, high cholesterol, cardiovascular disease and chronic kidney disease).

Supplemental Table S4. The joint relationship of tea consumption and milk consumption with risk of new-onset acute kidney injury (AKI)*.

Tea consumption, cups per day	Total	No of events	Adjusted Model 1†		Adjusted Model 2†	
			HR (95%CI)	*P value*	HR (95%CI)	*P value*
**Not consuming tea or milk**	6743	398	ref		ref	
**Only consuming milk**	66456	3233	0.78(0.70, 0.87)	<0.001	0.82(0.74, 0.91)	<0.001
**Only consuming tea**						
≤2	4439	200	0.67(0.57, 0.80)	<0.001	0.80(0.67, 0.94)	0.009
3-5	3573	161	0.60(0.50, 0.73)	<0.001	0.70(0.58, 0.84)	<0.001
6-8	1398	74	0.76(0.59, 0.97)	0.028	0.81(0.63, 1.04)	0.095
≥9	486	27	0.91(0.62, 1.35)	0.649	0.91(0.62, 1.35)	0.655
**Consuming both tea and milk**						
≤2	124701	5046	0.58(0.52, 0.64)	<0.001	0.72(0.65, 0.80)	<0.001
3-5	198033	7867	0.54(0.49, 0.60)	<0.001	0.68(0.61, 0.75)	<0.001
6-8	73369	3114	0.59(0.54, 0.66)	<0.001	0.69(0.62, 0.77)	<0.001
≥9	18995	1051	0.80(0.71, 0.90)	<0.001	0.79(0.71, 0.89)	<0.001

Adjusted Model 1: Adjusted for age, sex, and ethnicity; Adjusted Model 2: adjusted for the covariates in Model 1 and further adjusted for educational attainment, household income, employment, Townsend Deprivation Index, body mass index, smoking status, alcohol consumption, physical activity, healthy diet score, coffee consumption, water consumption, and comorbidities (hypertension, diabetes, high cholesterol, cardiovascular disease and chronic kidney disease).

Supplemental Table S5. HR (95%CI) for new-onset acute kidney injury (AKI) associated with tea consumption in various subgroups*.

	Tea consumption, cups per day					P for interaction
	0	≤2	3-5	6-8	≥8	
**Age, years**						0.214
<60	ref	0.83(0.77,0.90)	0.82(0.77,0.88)	0.82(0.75,0.89)	1.01(0.90,1.14)	
≥60	ref	0.90(0.86,0.95)	0.85(0.81,0.89)	0.87(0.82,0.92)	0.96(0.88,1.05)	
**Sex**						0.742
Female	ref	0.87(0.82,0.93)	0.83(0.78,0.88)	0.84(0.78,0.91)	1.03(0.92,1.15)	
Male	ref	0.86(0.81,0.91)	0.80(0.76,0.85)	0.82(0.77,0.88)	0.93(0.85,1.02)	
**Body mass index, kg/m^2**						0.058
<25	ref	0.82(0.74,0.91)	0.79(0.72,0.86)	0.85(0.76,0.94)	1.10(0.95,1.28)	
25-<30	ref	0.87(0.81,0.93)	0.81(0.76,0.86)	0.82(0.76,0.89)	0.98(0.88,1.10)	
≥30	ref	0.87(0.82,0.93)	0.82(0.77,0.87)	0.81(0.75,0.88)	0.86(0.77,0.96)	
**Hypertension**						0.375
No	ref	0.84(0.76,0.92)	0.83(0.76,0.91)	0.87(0.78,0.96)	1.00(0.87,1.16)	
Yes	ref	0.87(0.83,0.92)	0.81(0.77,0.85)	0.82(0.77,0.87)	0.95(0.88,1.03)	
**Diabetes**						0.359
No	ref	0.86(0.82,0.91)	0.80(0.76,0.84)	0.82(0.78,0.87)	0.96(0.89,1.04)	
Yes	ref	0.85(0.77,0.93)	0.85(0.78,0.92)	0.86(0.78,0.96)	0.93(0.80,1.08)	
**High cholesterol**						0.755
No	ref	0.87(0.82,0.92)	0.82(0.78,0.87)	0.85(0.80,0.91)	0.98(0.89,1.07)	
Yes	ref	0.87(0.81,0.93)	0.81(0.76,0.86)	0.81(0.75,0.87)	0.95(0.85,1.06)	
**Cardiovascular disease**						0.909
No	ref	0.86(0.82,0.91)	0.82(0.78,0.86)	0.83(0.79,0.88)	0.97(0.89,1.05)	

Yes	ref	0.88(0.80,0.97)	0.80(0.74,0.88)	0.81(0.73,0.90)	0.96(0.83,1.10)	
**Chronic kidney disease**						0.341
No	ref	0.86(0.82,0.91)	0.81(0.77,0.85)	0.85(0.80,0.90)	0.94(0.86,1.02)	
Yes	ref	0.87(0.79,0.95)	0.81(0.74,0.88)	0.80(0.73,0.89)	1.06(0.93,1.22)	
**Smoking status**						0.267
Never	ref	0.87(0.82,0.94)	0.82(0.77,0.87)	0.80(0.74,0.87)	0.99(0.88,1.12)	
Former	ref	0.87(0.81,0.93)	0.82(0.77,0.88)	0.87(0.81,0.94)	0.91(0.81,1.02)	
Current	ref	0.84(0.76,0.94)	0.77(0.70,0.84)	0.81(0.72,0.90)	1.01(0.89,1.15)	
**Alcohol consumption, times/week**						0.239
**<1**	ref	0.88(0.82,0.94)	0.85(0.80,0.91)	0.86(0.80,0.93)	1.00(0.91,1.11)	
≥1	ref	0.85(0.81,0.90)	0.78(0.74,0.83)	0.80(0.75,0.85)	0.93(0.85,1.02)	
**Optimal physical activity**						0.717
No	ref	0.90(0.84,0.96)	0.83(0.78,0.89)	0.85(0.79,0.92)	0.96(0.86,1.07)	
Yes	ref	0.84(0.79,0.90)	0.80(0.76,0.85)	0.82(0.76,0.88)	0.96(0.87,1.06)	
**Healthy diet score**						0.426
<4	ref	0.86(0.82,0.91)	0.80(0.76,0.84)	0.81(0.76,0.86)	0.96(0.88,1.05)	
≥4	ref	0.87(0.81,0.94)	0.84(0.79,0.90)	0.87(0.80,0.94)	0.97(0.86,1.09)	

*Adjusted, if not stratified, for age, sex, ethnicity, educational attainment, household income, employment, Townsend Deprivation Index, body mass index, smoking status, alcohol consumption, physical activity, healthy diet score, coffee consumption, water consumption, and comorbidities (hypertension, diabetes, high cholesterol, cardiovascular disease and chronic kidney disease).

Supplemental Table S6. Sensitivity analysis for the association between tea consumption and new-onset acute kidney injury*.

	Tea consumption, cups per day				
	**0**	**≤2**	**3-5**	**6-8**	**≥8**
**Sensitivity analysis 1: further adjustments for intensive care unit (ICU) admission, sepsis, viral infections, and bacterial infections before the end of follow-up**					
No. of participants	73287	129272	201748	74811	19503
No. of events	3638	5253	8040	3192	1079
Adjusted HR (95%CI) *	ref	0.89(0.85, 0.93)	0.88(0.84, 0.91)	0.88(0.84, 0.93)	0.96(0.89, 1.03)
**Sensitivity analysis 2: further adjustments for drug uses (cholesterol lowering medication, anti-hypertensive drug, antidiabetic, aspirin, ibuprofen, paracetamol, and diclofenac) at baseline**					
No. of participants	73287	129272	201748	74811	19503
No. of events	3638	5253	8040	3192	1079
Adjusted HR (95%CI) *	ref	0.86(0.83, 0.90)	0.81(0.77, 0.84)	0.82(0.78, 0.86)	0.94(0.87, 1.00)
**Sensitivity analysis 3: excluding 15314 participants with a previous history of migraines and censoring at new migraines diagnosis during follow-up**					
No. of participants	70875	125558	195463	72137	18785
No. of events	3512	5092	7752	3086	1036
Adjusted HR (95%CI) *	ref	0.87(0.83, 0.90)	0.81(0.77, 0.84)	0.83(0.78, 0.87)	0.94(0.88, 1.01)
**Sensitivity analysis 4: further adjustments for insomnia, and irritability**					
No. of participants	70875	125558	195463	72137	18785
No. of events	3512	5092	7752	3086	1036
Adjusted HR (95%CI) *	ref	0.87(0.83, 0.91)	0.81(0.78, 0.85)	0.83(0.79, 0.87)	0.95(0.88, 1.02)
**Sensitivity analysis 5: excluding 297 participants with a previous history of drug use disorders and censoring at new drug use disorders diagnosis during follow-up**					
No. of participants	73227	129214	201666	74753	19464

No. of events	3603	5215	7989	3171	1069
Adjusted HR (95%CI) *	ref	0.87(0.83, 0.91)	0.81(0.78, 0.85)	0.83(0.79, 0.87)	0.95(0.89, 1.02)
**Sensitivity analysis 6: further adjustments for lifetime cannabis use in a subsample who completed an online Mental Health Questionnaire**					
No. of participants	22577	43259	63603	22327	5150
No. of events	612	1008	1371	477	143
Adjusted HR (95%CI) *	ref	0.91(0.82, 1.00)	0.83(0.75, 0.92)	0.80(0.70, 0.91)	0.93(0.77, 1.12)
**Sensitivity analysis 7: restricting to a subsample who did not make any major changes to diet in the last 5 years**					
No. of participants	43009	79222	123519	44824	11528
No. of events	1862	2828	4218	1633	551
Adjusted HR (95%CI) *	ref	0.88(0.83, 0.94)	0.81(0.76, 0.86)	0.81(0.76, 0.87)	0.95(0.86, 1.05)

*Adjusted for age, sex, ethnicity, educational attainment, household income, employment, Townsend Deprivation Index, body mass index, smoking status, alcohol consumption, physical activity, healthy diet score, coffee consumption, water consumption, and comorbidities (hypertension, diabetes, high cholesterol, cardiovascular disease and chronic kidney disease).
